# An emerging view on vascular fibrosis molecular mediators and relevant disorders: from bench to bed

**DOI:** 10.3389/fcvm.2023.1273502

**Published:** 2023-12-21

**Authors:** Rongxuan Hua, Han Gao, Chengwei He, Shuzi Xin, Boya Wang, Sitian Zhang, Lei Gao, Qiang Tao, Wenqi Wu, Fangling Sun, Jingdong Xu

**Affiliations:** ^1^ Department of Clinical Medicine, School of Basic Medical Sciences, Capital Medical University, Beijing, China; ^2^ Department of Clinical Laboratory, Aerospace Center Hospital, Peking University, Beijing, China; ^3^ Department of Physiology and Pathophysiology, School of Basic Medical Sciences, Capital Medical University, Beijing, China; ^4^ State Key Laboratory of Holistic Integrative Management of Gastrointestinal Cancers, Beijing Key Laboratory of Carcinogenesis and Translational Research, Peking University Cancer Hospital & Institute, Beijing, China; ^5^ Department of Biomedical Informatics, School of Biomedical Engineering, Capital Medical University, Beijing, China; ^6^ Experimental Center for Morphological Research Platform, Capital Medical University, Beijing, China; ^7^ Department of Experimental Animal Laboratory, Xuan-Wu Hospital of Capital Medical University, Beijing, China

**Keywords:** vascular fibrosis, ECM, TGF-β, RAAS, arterial stiffness, CVD

## Abstract

Vascular fibrosis is a widespread pathologic condition that arises during vascular remodeling in cardiovascular dysfunctions. According to previous studies, vascular fibrosis is characterized by endothelial matrix deposition and vascular wall thickening. The RAAS and TGF-β/Smad signaling pathways have been frequently highlighted. It is, however, far from explicit in terms of understanding the cause and progression of vascular fibrosis. In this review, we collected and categorized a large number of molecules which influence the fibrosing process, in order to acquire a better understanding of vascular fibrosis, particularly of pathologic dysfunction. Furthermore, several mediators that prevent vascular fibrosis are discussed in depth in this review, with the aim that this will contribute to the future prevention and treatment of related conditions.

## GRAPHICAL ABSTRACT

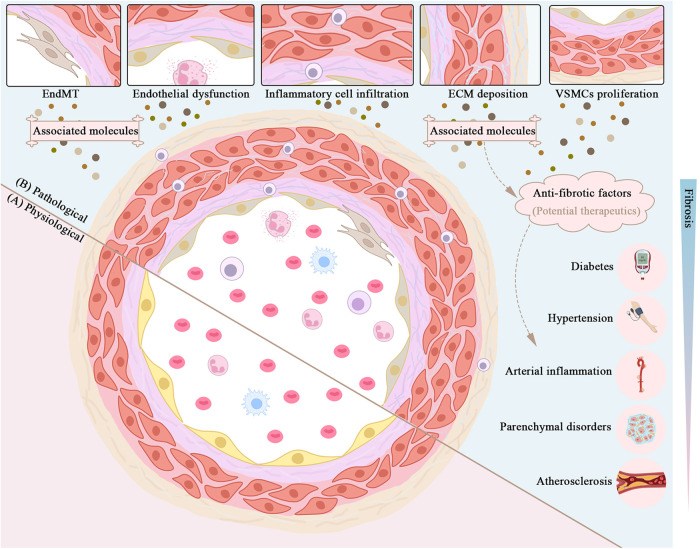

## Highlights

• Endothelial-to-mesenchymal mesenchymal transition, smooth muscle cell proliferation, and extracellular matrix deposition are all symptoms of vascular fibrosis.
• Progressive vascular fibrosis is tied closely to multisystem diseases including hypertension, atherosclerosis, and diabetes.
• Multiple newly discovered variables, including the renin–angiotensin–aldosterone system and transforming growth factors-β, contribute to vascular fibrosis.
• Inflammation and mitochondrial damage can both contribute to vascular fibrosis.

## 1. Introduction

The vascular system is made up of numerous arteries, capillaries, and veins, and is found throughout the body, from organs to tissues. Its major role is to keep the blood flowing, which is required for cell growth, nutrition absorption, metabolite transfer, and hence intracellular stability (1). However, changes in the vascular anatomy can bring crucial disruption to the homeostasis in vascular function. Vascular fibrosis is one of the most prevalent biochemical abnormalities in the cardiovascular system. More than 60% of individuals over the age of 70 have arterial stiffness (2), which can be aggravated by vascular fibrosis (3). Initially, vascular fibrosis begins as a reversible compensatory adaptation process. Persistent stimulation of the renin–angiotensin–aldosterone system (RAAS), inflammation, oxidative stress (OS), and other factors might result in irreversible vascular fibrosis due to myofibroblast activation and extracellular matrix (ECM) deposition (1). Their harsh impacts would spread from blood vessels into parenchymal tissue, triggering reactions in a variety of systems and organs, for instance, the heart, brain, stomach, kidneys (3). In this review, we outline the structure and function of the vascular system, lay stress on the processes and mediators of vascular fibrosis, and highlight some therapeutic strategies for associated conditions. We also analyze the most recent research methodologies and advances in vascular fibrosis, meanwhile bringing different theoretical foundations and approaches for future clinical trials.

Box 1. **Pathogenic shifts during vascular fibrosis**

Vascular fibrosis is the overgrowth, hardening, scarring of blood vessels, which is attributed to excess deposition of ECM. Many scholars believe there are four major pathogenic variables that cause vascular fibrosis: (i) Fibroblasts as the primary cell involving in the production and deposition of ECM proteins in the vascular wall. Changes in genes that express distinct collagen subtypes in fibroblasts effect fibrotic process (4). (ii) ECM proteins accumulation is secondary to fibroblast changes, and can be mediated by plenty of molecules or events, such as TNF-α, IL-6, IL-1, and OSM. (iii) TGF-β action appears to be dictated by its biological source, with macrophage originated TGF-β typically demonstrating wound-healing and profibrotic activity, while Treg cells produced TGF-β operates as an anti-inflammatory and antifibrotic mediator (5). (iv) Endothelial cells release cytokines and adhesion molecules which elevate the inflammatory and potential fibrotic response, in response to wounded tissues and activated immune cells (6).

## 2. Vascular structure and function: from physiology to fibrosis

Under physiological conditions, blood vessels behave as more than simply a pipeline for blood transport, but as an organ capable of adjusting their diameter to regulate blood pressure elastically or muscle-dependently. In the circulatory system, blood flows from the left ventricle to the artery, which then branches into arterioles and connects with venules through capillaries, before returning to the right atrium.

Notwithstanding the circulatory system's heterogeneity and mutability, the histological arrangement is indeed very comparable. Except for capillaries, blood vessels are composed of three layers: tunica intima, tunica medium, and tunica adventitia (7), in which ECM is their mutual internal environment for cell survival. This critical component is filled with numerous proteins including elastin fibers, collagen (Col), fibronectin (FN), proteoglycans, and others (8), making it indispensable in transducing cellular signaling and cellular interactions, as well as connecting all the cells in vascular wall.

**Figure 1:**
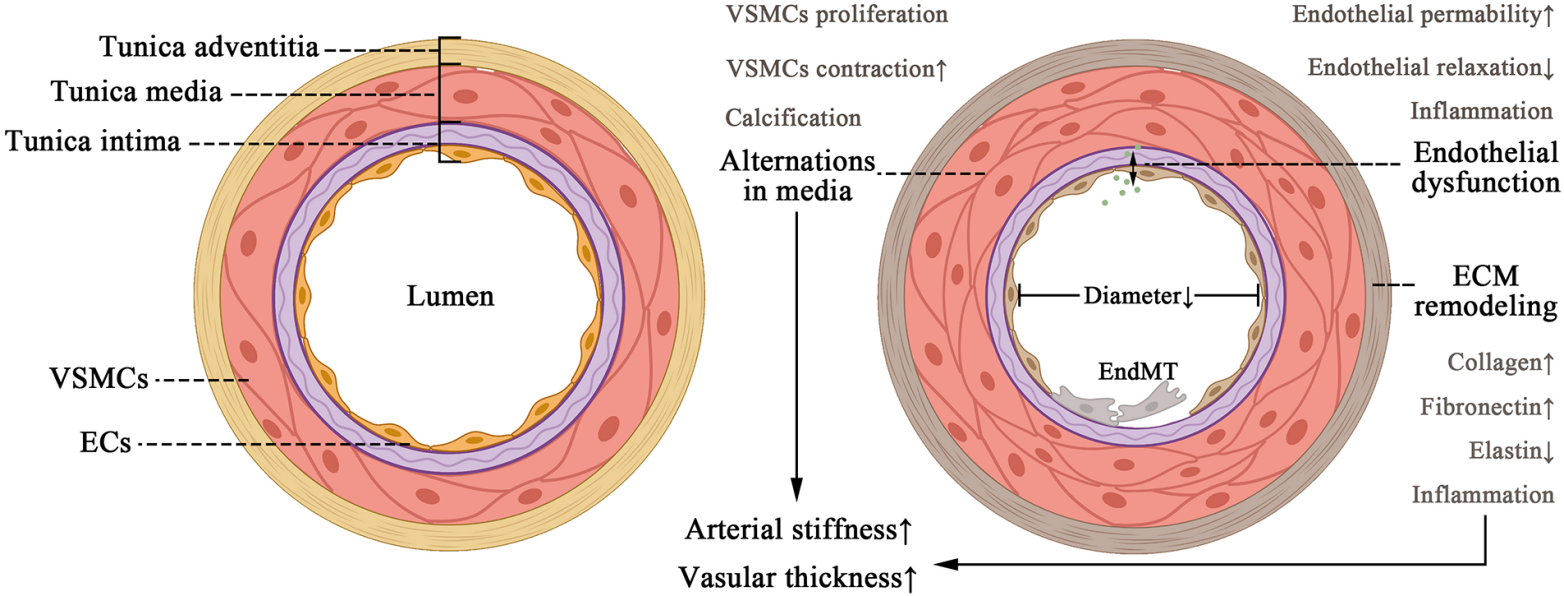
Pattern of changes in vascular structural composition (left) and alternations in a pathological fibrosing state (right). The tissue structure of the vessel wall, including tunica intima, media, and adventitia. The appropriate contraction of VSMCS and the amount of ECM protein ensure the normal elastic function of the vessel under physiological conditions. As vascular fibrosis develops, it leads to endothelial dysfunction, ECM remodeling, and EndMT, leading to arteriosclerosis and thickening of the vessel wall ultimately.

The intima, the thinnest of the three layers, is consisted of a single layer of endothelial cells (ECs) that make direct contact with the lumen. Therefore, EC is taking on a crucial role in cellular homeostasis, such as vascular tone modulation, coagulation and anticoagulation, and immune response (7). Antigen presentation characteristics in EC were discovered due to a coating of glycocalyx on its surface (9). However, since ECs have such high biological activity, plasma antigens or chemicals may trigger specific clinical disorders. A sub-endothelial layer packed with fibro elastic connective tissue lies beneath the ECs, stabilizing them yet enabling them to be flexible (7). Another type of cell that should not be overlooked is Pericyte. They can differentiate into ECs, fibroblasts, vascular smooth muscle cells (VSMCs), adipocytes, and osteoblasts and eventually contribute in blood flow dynamics modulation (10, 11). In most cases, vascular injury begins with intima damage induced by hemodynamics or morbific materials in the circulation (Figure 1).

The media is thicker than the intima and adventitia. It is composed of a significant number of VSMCs and well-ordered elastin fibers that act as vessel dimension regulators (12). The adventitia is the most complicated part of the arterial wall since it comprises not only a considerable amount of fibro-elastic connective tissue, but also a diversity of cell phenotypes such as fibroblasts, pericytes, and immune cells (13) (Figure 1).

Unfortunately, once fibrosis worsens, substantial abnormalities in the structure of blood vessels emerge, leading to perfusion dysfunction in systemic circulation and organs. ECM deposition and media hypertrophy lead to thickening of the vascular wall and reduction in elasticity, which terminally contribute to hemorheological abnormalities (2). During vascular fibrosis, the media augmented VSMC contraction and calcification (1). Meanwhile the adventitia experiences remodeling (14), and the intima undergoes endothelial dysfunction due to decreased endothelial relaxation, increased permeability, and inflammation (15). These alterations in endothelium are associated with vascular fibrosis and sclerosis, illustrating its importance as a physiologic barrier against circulating immune cells and inflammatory cells. However, recent research has revealed that cytokines can initiate EC to secrete pro-inflammatory and profibrotic molecules, thereby facilitating inflammation and fibrosis in endothelial dysfunction (16). EC to mesenchymal transition (EndMT) also represents multiple cardiovascular disorders (CVD), fibrosis included (16), once more emphasizing the role of endothelial cells (Figure 1). So far, we have elaborated a histological and functional feature in vascular fibrosing process, which is of great significance in understanding what we discuss later.

## 3. Molecular mechanisms of vascular fibrosis

Vascular fibrosis is caused mainly by EC dysregulation, VSMC proliferation, fibroblast activation, ECM remodeling, and immune cell infiltration. In this section, we highlighted some molecules of concern in cardiovascular pathophysiology and categorized them in Table 1. Propelling the understanding on underlying processes and associated molecules will provide a number of strategies for treating or alleviating sickness.

**Table 1:** Summary of pro-fibrotic and anti-fibrotic molecules discussed in this review.

Category	Mediator	Pro-fibrotic or Anti-fibrotic	Influence	Notes	References
Canonical	Ang II	Pro-fibrotic	ECM deposition (collagen & fibronectin overexpression, elastin reduction) and VSMC proliferation	via AT-1R	(1, 2, 17)
		Anti-fibrotic	Vasodilation, anti-inflammation and growth inhibition	via AT-2R	(18, 19)
	Aldo	Pro-fibrotic	ECM deposition (collagen & fibronectin overexpression, elastin reduction) and VSMC proliferation	via MR	(17, 20, 21)
	Ang-(1-7)	Anti-fibrotic	Vasodilation, anti-inflammation, and Ang II inhibition	ACE2-Ang-(1-7)/MasR axis	(22–24)
	TGF-β	Pro-fibrotic	ECM deposition (collagen & fibronectin overexpression, elastin reduction) and VSMC proliferation	via Smad2/3/4, RhoA/ERK, p38/MAPK, JAK/STAT, PI3K/AKT, Galectin-3, IL-11, etc.	(25–29)
	MMP	Pro-fibrotic	Degrading collagen & elastin	Imbalance in MMP family is the cause of ECM deposition	(1, 30, 31)
	TIMP	Anti-fibrotic	Inhibiting MMP	/	(30)
	TG 2	Pro-fibrotic	ECM deposition (collagen secretion & scaffolding ECM proteins)	/	(17, 32, 33)
	CTGF	Pro-fibrotic	ECM deposition	/	(2, 30)
	FGF	Pro-fibrotic	ECM deposition	/	(2, 30)
Non-canonical	Fetuin-A	Anti-fibrotic	Suppressing TGF-β signaling	Inhibiting Smad3 phosphorylation	(34)
	Salusin-β	Pro-fibrotic	Promoting TGF-β signaling, increasing Col-1/3, FN, TGF-β and CTGF expression	Stimulating Smad2/3 phosphorylation	(35)
	Alamandine	Anti-fibrotic	Attenuating Ang II-induced col-1, TGF-β and CTGF overexpression	via stimulating MrgD expression and inhibiting p38 phosphorylation	(36)
	Galectin-3	Pro-fibrotic	Increasing Col-1 deposition, MMP-9 decrease, VSMC proliferation	Increased by multiple factors (inflammation, ROS, TGF-β, aging)	(27, 37, 38)
	IL-11	Pro-fibrotic	Increasing collagen & MMP (via ERK)	Downstream molecule of TGF-β; X203 alleviates vascular fibrosis	(28, 29)
	StAR	Pro-fibrotic	Increasing Aldo	Downstream signal of c-Fos/c-Jun	(39)
	BMS	Anti-fibrotic	Decreasing collagen deposition & media thickness	ET-1 antagonist	(40)
Energy metabolism in mitochondria	Mitochondria OS	Pro-fibrotic	Increasing Col-1/3, CTGF, TGF-β expression & media thickness	Along with ER stress	(41–43)
	HFD	Pro-fibrotic	ECM deposition (Col-1/3, CTGF expression) and vascular thickness	/	(44)
	MitoQ	Anti-fibrotic	Inhibiting mitochondria OS	Alleviating Ang II-induced fibrosis	(44–46)
	FABP3	Pro-fibrotic	ECM deposition (Col-1, FN-1, MMP3/9 overexpression)	FABP3 deficiency is also unfavorable	(47, 48)
EndMT	EndMT	Pro-fibrotic	Increasing collagen expression; NO shortage; ROS accumulation; inflammation	Increased by multiple factors (IL-1, IL-6, TNF-α, IFN-γ, ET-1, etc.)	(49–52)
	let-7	Anti-fibrotic	Reversing EndMT	Forming TGF-βR1/let-7i double-negative feedback pathway	(53)
	H_2_S	Anti-fibrotic	Suppressing TGF signal & RAAS; increasing NO; inhibiting CD11b + leukocytes; inhibiting OS	Blocker of EndMT	(54–57)
Immune cells and inflammation	IL-17	Pro-fibrotic	Increasing gal-3, collagen deposition, ROS accumulation, fibroblasts	Increased by Aldo via MR	
	Caspase-1	Pro-fibrotic	Increasing Col-1 & FN expression	Pyroptosis related	(58–60)
	Macrophage autophagy	Anti-fibrotic	Inhibiting M1 polarization; suppressing EndMT;	/	(61–63)
	Macrophage polarization	Pro-fibrotic	Dual stimulation between pro-inflammatory (IL-1β, IL-6, TNF-α, ROS, NO) molecules and anti-inflammatory (IL-4, IL-10, IL-13) molecules	Imbalanced M1/M2 ratio cause chronic inflammation in fibrosis	(62–65)
	IL-17A	Pro-fibrotic	Increasing ROS, endothelial dysfunction, fibroblasts proliferation; NGAL&PYK2 expression upregulation	Can be expressed in response to Aldo signal	(66, 67)
	PTEN	Anti-fibrotic	Decreasing TGF-β, Col-1, FN-1, MMP-2 expression	Preventing pyroptosis and apoptosis through PTEN/PI3K/AKT axis	(68–70)
	Galectin-3	Pro-fibrotic	Increasing IL-1 & IL-18 expression and inflammatory cell extravasation	Inducing NF-κB pathway	(71–73)
	EVs	Pro-fibrotic	EVs form different sources can carry different pro-fibrotic cargos (miR-23a-3p, lncRNA GAS5, circUbe3, miR-142-3p, etc.)	The pro-fibrotic feature is shown by molecules within EVs, not EVs themselves	(74–77)
	miR-21	Pro-fibrotic	Regulating inflammation via PPAR-α; TGFβ1/Smad3 pathway	Has anti-inflammatory effect	(78, 79)
	miR-122	Pro-fibrotic	Inhibiting PTEN; inducing OS, apoptosis, and inflammatory response	/	(80)
Others	MBG	Pro-fibrotic	Promoting TGF-β signaling; suppressing Fli-1	Being an endogenous cardiotonic steroid	(81, 82)
	Fli-1	Anti-fibrotic	Inhibiting Col-1 expression	Being a nuclear inhibitory transcription factor	(81, 82)
	spironolactone	Anti-fibrotic	Suppressing MBG's effects	/	(83)
	ROCK	Pro-fibrotic	Promoting VSMC activation, inflammation, OS, NO production and EndMT	Can be suppressed by statins	(84–86)
	PAI-1	Pro-fibrotic	Increasing Col-1 expression; inducing Rho kinase activation	Increased by Ang II; can be suppressed by apelin	(87, 88)
	apelin	Anti-fibrotic	Inhibiting Ang II-induced PAI-1, collagen and MMP-2 expression	Can be suppressed by miR-122	(88)

### 3.1. Renin–angiotensin–aldosterone system and TGF-β induce profibrotic changes in ECM

RAAS and TGF-β are the canonical regulators in vascular fibrosis. Although they are not new to public attention, it is still necessary to be elaborated for the better understanding of newfound evidences. Initially, studies on RAAS concentrated on hypertension and heart failure, but later shifted to vascular fibrosis. TGF-β has been identified as an essential mediator in fibrotic parenchymal organs. In the case of vascular fibrosis, however, a linkage between RAAS and TGF-β is revealed.

#### 3.1.1. RAAS

**Figure 2:**
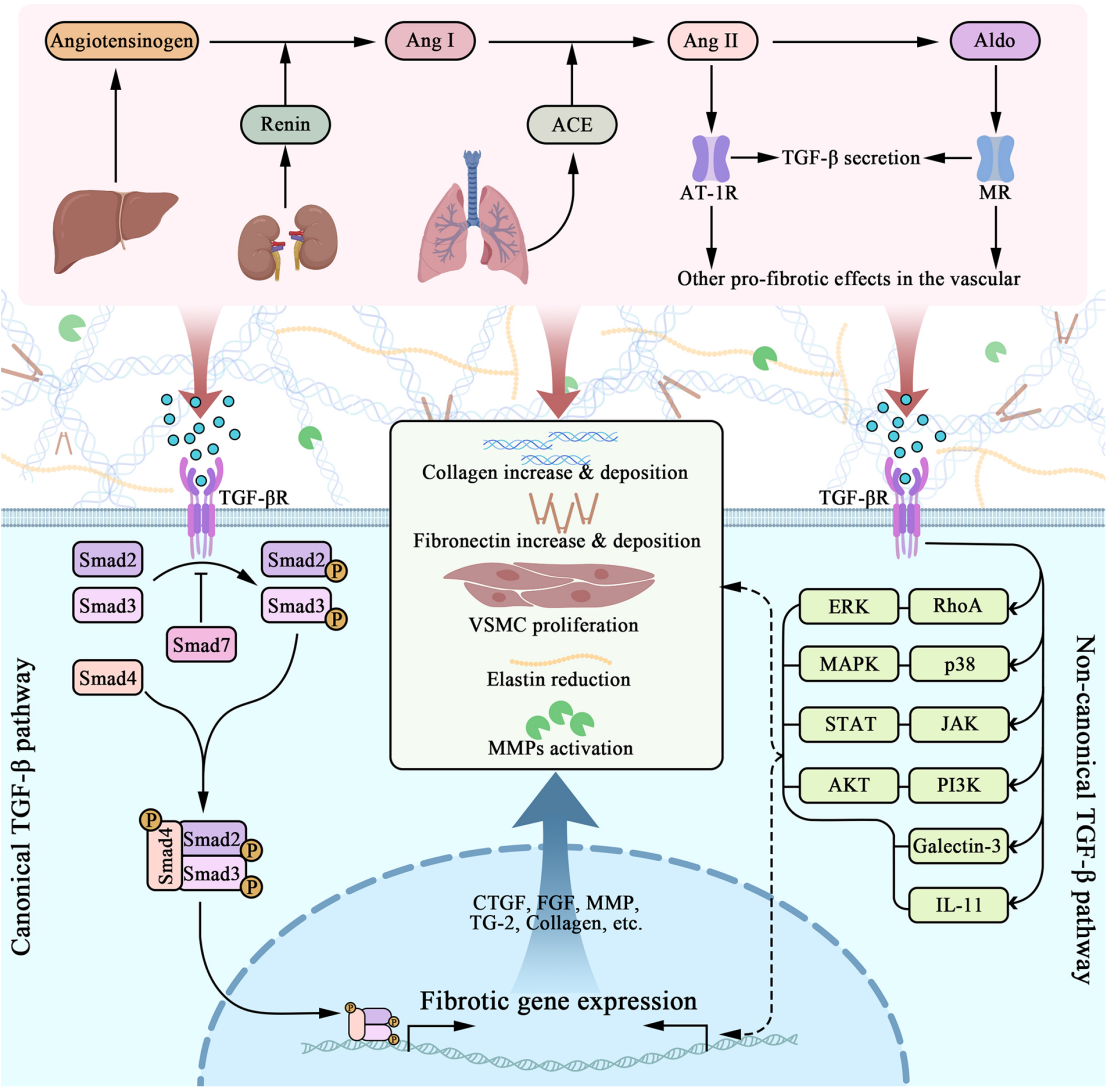
Molecular pathways of RAAS and TGF-β interactively induced vascular fibrosis. RAAS contains a series of molecules that are orderly activated, including angiotensinogen, renin, ACE, angiotensin I and II, and aldosterone. Among them, Ang II and Aldo plays crucial role in promoting fibrosis, acting through AT-1R and MR respectively. As a consequence, vascular cells gain a pro-fibrotic phenotype that includes TGF-β secretion. TGF-β acts through the cell membrane, triggering several intracellular molecular pathways, among which Smad signaling was canonically known. TGF-β/Smad induces nuclear fibrotic gene expression, followed by ECM, as well as VSMC changes, including collagen overexpression, elastin reduction, collagen and fibronectin deposition, MMP activation (along with MMP/TIMP imbalance) and so on. Progressively, preceding alternations directly lead to vascular fibrosis.

Renin, also known as angiotensinogenase, is a kidney-secreted enzyme that catalyzes angiotensin II (Ang II) synthesis on its substrate via angiotensin-converting enzyme (ACE), meanwhile Ang II can further stimulate aldosterone (Aldo) secretion (20). RAAS is essential to water-sodium retention and vasopressor function, therefore being one of the most highly considered mediator in cardiovascular system. Intriguingly, previous studies indicated that Ang II and Aldo have an influence on developing vascular fibrosis, respectively via Ang II receptor 1 (AT-1R) and mineralocorticoid receptor (MR). These signals subsequently upregulates TGF-β expression in ECs, VSMCs, fibroblasts, and macrophages (17) (Figure 2). Additionally, activated AT-1R and MR is also responsible to EC/VSMC dysfunction, OS, macrophage activation, dysregulation of autophagy, during fibrosis in vascular pathology (17, 21, 89, 90). It is noteworthy that AT-1R triggers vasoconstriction, VSMC proliferation, inflammation, and extracellular matrix remodeling, whereas AT-2R activation have completely contradictory effects and often represents protection in blood vessels (18). In fact, scholars have found AT-2R exhibits vasodilation through regulating production of bradykinin, NO, and cGMP (19). Meanwhile, decreased TNF-α-mediated IL-6 secretion via inhibiting NF-κB activation and reducing MAPK activity are also the ways how AT-2R respectively manage anti-inflammation and growth inhibition (19). Additionally, research found AT-2R can significantly attenuate the expression of TGF-β type I receptor in VSMCs of WKY rat (91). Recent researches claim that AT-2R agonists have astonishing potential in clinical treatment, for instance, Compound 21 are now in phase III clinical trial (92, 93). Still, few studies have focused their significance into vascular fibrosis.

However, there are two opposing arms in RAAS, and above-discussed ACE/Ang II/AT1R axis refers to a pro-fibrotic, pro-inflammatory “classical arm”. The “protective arm”, though not dominant, have essential role in counteracting negative effects in “classical arm” via ACE2/Ang-(1-7)/MasR axis (22). ACE2 as a homolog of ACE, converts Ang II to Ang-(1-7) through a carboxypeptidase feature, therefore inhibits Ang II by degradation (94). Furthermore, Ang-(1-7) can be formed in an Ang II alternative way, by firstly synthesizing Ang-(1-9) from Ang I via ACE2, then transforming into Ang-(1-7) via ACE (23). Ang-(1-7) then activates its specific Mas receptor (MasR) in EC, which increases the production of vasodilators such as nitric oxide (NO) and prostacyclin (24). In addition to vasodilation, MasR is also involved in many vascular protective functions, including anti-fibrotsis, anti-inflammation, and anti-proliferation (22). Meanwhile, Ang-(1-7) has been reported to downregulate TGF-β1/Smad2 level in rabbits after angioplasty (95). Therefore, multiple researches have been attracted to the therapeutic potential that ACE2/Ang-(1-7)/MasR axis possesses in cardiovascular field.

Accumulating research found that treatment with human recombinant ACE2 (hrACE2) constrains the cardiac fibrosis, OS, and inflammation that Ang II induced (96). A small molecule ACE2 activator, XNT, reduced hydroxyproline accumulation in kidney and myocardium (97). Furthermore, diminazene aceturate (DIZE) as a ACE2 activator and AT1R inhibitor, shows alleviation to collagen accumulation in cardiac infraction mice (98). Besides ACE2 activators, researchers recently found a nonpeptide, Ang-1(1-7)-like compound named AVE 0991 that can selectively activates MasR and is of great potential as cardiovascular medicine (99). However, more evidences are needed when it comes to vascular fibrosis.

#### 3.1.2. TGF-β

TGF-β literally act as an intersection, mediating a series of downstream pro-fibrotic signaling pathway that eventually promote ECM protein reconfiguration and VSMC proliferation, while receiving multiple upstream mediators that modulate the preceding process. Through decades of researching, evidences suggest TGF-β induces Smad (small mothers against decapentaplegic)-2/3/4 phosphorylation, which results in further profibrotic gene expression (25). Yet, not all Smad are pro-fibrotic, Smad-7 has been proved to decrease Smad-2/3 phosphorylation (25). Intriguingly, scholars have explicated that Smad signal can be triggered independently to TGF-β (100). Study have shown that Ang II-mediated ECM synthesis can be managed by activation of PKC and p38 MAPK, triggering NF-κB and Smad2/3 cross-talk pathway, which is independent to TGF-β (100). It is supported by further study that Ang II-induced Smad2/3 nuclear translocation and DNA-binding activity via AT-1R have not been dissolved by blockade of endogenous TGF-β (101). Besides TGF-β/Smad, many studies have suggested several other pathways that are as well involved in TGF-β signaling, including galectin-3, IL-11, RhoA/ERK, p38/MAPK, JAK/STAT and PI3K/AKT (26) as depicted in Figure 2.

Noteworthy, there are plenty of TGF-β interacting molecules with great importance. To begin with, fetuin-A is a serum glycoprotein produced by liver, and can suppress TGF-β1 signaling by upregulating pseudoreceptor of TGF-β1 (34). A study among 106 participants found a negative connection between serum fetuin-A levels and peripheral artery disease in atherosclerosis (102). In vitro, fetuin-A stimulation can significantly relieve TGF-β1-induced overexpression of TGF-β1 and Col-1 (34). Secondly, salusin-β is a bioactive peptide that participates in CVD. Recent study indicates their part in promoting vascular fibrosis through TGF-β1/Smad and VSMCs proliferating (35), as well as Col-1/3 overexpression, CTGF mRNA upregulation and Smad2/3 phosphorylation (35). Additionally, a RAAS component named alamandine can attenuate Ang II-induced elevation of Col-1, TGF-β and CTGF, alleviating vascular fibrosis via MrgD (*Mas*-related G protein-coupled receptor member D) expression (36). Galectin-3 (gal-3) is another TGF-β interactive molecule that worth special noticing. It has lately been explored intensively throughout many studies, for it contributes to multiple cardiovascular fibrotic pathways (37). Gal-3 expresses in fibroblasts, ECs, and immune cells, causing Col-1a deposition and MMP (matrix metalloproteinases) reduction, in response to TGF-β1 signaling via STAT-3 (27). This process can be reversed by gal-3 siRNA (38). Meanwhile, a univariate analysis found gal-3 being correlated with carotid intima-media thickness, arterial stiffness, cardiac output, indicating gal-3 is a favorable marker of cardiovascular function and fibrosis (103). Last but not least, recent studies have demonstrated that IL-11 (Interleukin 11), as the downstream molecule of TGF-β, triggers fibrosis along with ET-1 and PDGF in an ERK-dependent way (28, 29). It can also activate fibroblasts via IL-11RA (28). Meanwhile, a study on the IL-11 antibody, X203, revealed its efficacy in MMP and collagen downregulation, as well as VSMCs reduction *in vivo* (29). It is worth emphasizing that IL-11 intervention may be more advantageous than TGF-β, for TGF-β has pleiotropic roles, suppressing which may result in certain physiological dysfunction (28).

#### 3.1.3. ECM accumulation

ECM deposition is the ultimate lesion in vascular fibrosis, in response to all molecular signals that we discuss. Collagen and elastin fibers are the two most important ECM proteins. In particular, their balance and their absolute amount determines the structure and mechanical properties of the blood vessel. It is of great significance to stress that ECM proteins can be regulated by an endopeptidases family named matrix metalloproteinases (MMPs). There lies multiple factors that can regulate MMP expression, including RAAS related molecules (Ang II, TGF-β, Aldo, ET-1, etc.), pro-inflammatory cytokines (IL-6, TNF-α, IL-1, etc.), and reactive oxygen species (ROS) (1, 17, 30). However, it is believed that different MMP isoforms function diversely on ECM proteins, resulting in different fibrotic outcomes. For instance, MMP-2/9 participated in TGF-β1/Smad-induced myofibroblasts activation and monocytes/macrophages infiltration (31), yet MMP-1 overexpression alleviates fibrosing (104). Upregulation of MMP-2/9 causes collagen accumulation, whereas MMP-8/13 degrades collagen, which is hazardous in arterial plaque rupture (105, 106). Apart from MMPs, an endogenous inhibitor called TIMP (tissue inhibitor of metalloproteinase) is also essential in ECM remodeling. TIMP are inhibitive to MMPs, which echoes the fact that imbalance in MMP/TIMP frequently accompanies with vascular fibrosis (30).

Transglutaminase 2 (TG2), another ECM associated proteinases that acts as a crosslinking protein as well as an ECM scaffold enzyme, promotes fibrosis by acting on fibronectin, collagen, and laminin (17, 32). As research deepens, some researchers have revealed that TG2 participates in TGF-β1/Smad signaling for collagen synthesis and secretion, causing VSMCs proliferation and ECM remodeling in vascular fibrosis (33). According to earlier research, preceding ECM changes are as well related to the overexpression of connective tissue growth factor (CTGF), fibroblast growth factor (FGF), platelet-derived growth factor (PDGF), vascular endothelial growth factor (VEGF) (2, 30) (Figure 2). Furthermore, there are additional molecules that worth notice. BMS, an ET-1 antagonist, is beneficial in reducing collagen deposition and medium thickness (40). StAR (steroidogenic acute regulatory protein) is a downstream factor of the steroidogenic acute regulatory protein c-Fos/c-Jun, which leads to hypoxia-induced upregulation of ECM proteins including CTGF, Col-3, MMP-2/9 (39).

From the above experimental evidence, we can conclude that RAAS and access TGF-β play an important role in the induction and promotion of extracellular matrix. Meanwhile, there are complex and extensive universal links between them, therefore they are important targets for therapeutic intervention in fibrosis.

### 3.2. Energy metabolism dysfunction and oxidative stress in mitochondria as prerequisite in vascular fibrosis

According to research findings, mitochondrial oxidative stress is increased in cardiac remodeling and failure (107), and is highly linked to inflammation and fibrosis (41). It is well acknowledged that OS is characterized by an overabundance of ROS, which can be generated by aberrant mitochondrial metabolism and lead to mitochondrial malfunction. Mitochondrial DNA is extremely vulnerable since oxidative damage may set off a vicious cycle of ROS generation (42). Ang II has also been demonstrated to promote mitochondrial dysfunction in vascular endothelial cells, through increasing ROS production (43). Besides, mitochondrial dysfunction interacts with TGF-β on various levels to promote or inhibit tissue fibrosis (108). Histological and western-blotting studies show that high-fat diet (HFD) animals had an increase in cardiac and aortic fibrosis biomarker genes such as Col-1/3, CTGF, and TGF-β, as well as aortic media and carotid intima media thickness (44, 109). It is noteworthy that aforementioned abnormalities can be reversed by MitoQ, a mitochondrial antioxidant (44, 45), which partly contributes to the of OS induces fibrosis.

On the other hand, ER stress is associated to mitochondrial OS. ER stress protein markers, BiP and PDIA6, was also found increased in HFD mice and positively correlated with cardiac interstitial fibrosis (44). Luckily, MitoQ can relieve those variations. Further experiments on fibroblasts *in vitro* suggest the same, for that MitoQ and ER stress inhibitor (4-phenyl butyric acid) alleviates Ang II induced fibrotic alternations (44). Besides, research on human umbilical vein endothelial cells have also shown that Aldo suppresses the expression of SOD2 protein, mitochondria DNA and protein through MR. Intriguingly, another mitochondrial antioxidant (Mito-TEMPO) can also twist this attenuation (46). These evidences further indicate OS is taking part in fibrosis in an ER stress-dependent manner, and mitochondrial OS antagonists like MitoQ and Mito-TEMPO possess their therapeutic benefits. Besides, scholars discovered that particulate matter 2.5 (PM2.5) exposure triggers aortic fibrosis via mitochondrial OS with the mechanisms mentioned above (45).

Mitochondria serve as the cell's energy factory, in which fatty acid binding protein 3 (FABP3) subtly modulates the process of fatty acid oxidation (FAO) (110). FAO generates ATPs as an energy source during fibrosis, for VSMC proliferation, ECM protein secretion, and immune cell functions (110). Interestingly, recent research reveals that FABP3 elevation induces aortic adventitia fibroblasts growth and ECM deposition (47), hence contributing to vascular fibrosis. Immunohistochemistry data demonstrate a substantial relationship between the expression of FABP3 and adventitia ECM proteins, including Col-1, FN-1, MMP-3/9 (47). However, another research on FABP3 suggests the opposite. FABP3^−/−^ mice exhibited severer cardiac hypertrophy than wildtype mice owing to FAO dysfunction, which enhances glycolysis and toxic lipid accumulation (48). As a conclusion, neither elevation nor deficiency in FABP3 is beneficial, meaning that mitochondrial energy metabolism has deeper mechanism that merits further investigation.

Last but not least, emerging studies have corroborated that mitophagy effects mitochondrial dysfunction in organs and tissues fibrosing (111). Consequently, there emerges plenty of potential therapeutic targets, such as targeting mitophagy, miR-30a, mTORC1/PGC-1 axis, and targeting mitochondrial quality control (108, 112, 113). These targets have the potential to improve mitochondrial function and reduce oxidative stress, thereby slowing or reversing the accumulation of ECM. Besides, employing wide tactics of genetic engineering, small compounds targeting mitochondria, and mito-therapy are all therapeutic options.

### 3.3. Endothelial to mesenchymal transition in vessel as initial fibrosis

EndMT, as mentioned above, is pathological condition of endothelium that results in vascular fibrosis. It occurs when ECs cease to secrete their particular biomarkers, but instead products of mesenchymal and myofibroblasts like Col-1 and α-SMA (49). The problem is that EndMT not only manifests a dysfunction of endothelium integrity and altering to a migratory form, but also turns ECs into invasive and proliferative phenotype, all of which are contributive to fibrosing. Multiple signaling pathways are involved in the formation of EndMT, for instance, IL-1β, IL-6 and TNF-α can be triggers of TGF-β pathways via NF-κB (50). Meanwhile, IFN-γ acting through JAK/STAT pathway and ET-1 participation in TGF-β/Smad signaling as well promotes EndMT process (51, 52). Unfortunately, EndMT causes certain dysfunctions in vascular system. For instance, NO production in ECs was essential to maintaining integrative structures and functions of vessels, whereas growing EndMT causes NO shortage. Besides, EndMT also leads to vascular fibrosis related ROS accumulation, vascular inflammation and ECM deposition. Furthermore, evidence manifests that ischemia-reperfusion injury in particular, can be the direct trigger of EndMT, which in turn promotes atherosclerosis and arteriosclerosis, especially in ischemic stroke in brain (53). However, a circulating human microRNA family named let-7 has been identified to play crucial role in remodeling the blood-brain barrier and reversing EndMT caused by ischemic stroke via a TGF-βR1/let-7i double-negative feedback pathway (53).

Recently, hydrogen sulfide (H_2_S), an essential mediator, generates from L-cysteine through enzymatic reactions, has been established as a blocker of EndMT and vascular fibrosis process. There are currently four major approaches. Initially, H_2_S acts as a vasodilator by inhibiting the TGF-β/cGMP-PKG pathway and lowering RAAS (54, 114), especially in chronic hypertension-induced vascular alteration. Secondly, H_2_S can boost endothelial nitric oxide synthase (eNOS) for more sufficient NO synthesizing in guarding against EC dysfunctions and reducing blood pressure (55). Additionally, H_2_S also has an anti-inflammatory impact by blocking the migration of CD11b^+^ leukocytes (56). Finally, H_2_S has been demonstrated to be a negative regulator of OS, which is thought to be connected to EndMT (57). The foregoing facts have surely pushed H_2_S's function into the future treatments for vascular fibrosis.

### 3.4. Inflammation associated cellular and molecular changes in fibrosis

Inflammation is a key factor in the development and progression of fibrosis. Inflammatory cells and mediators are playing a crucial role in myofibroblast activation and ECM deposition. Understanding the cellular and molecular mechanisms of fibrosis may lead to the development of new therapeutic interventions.

#### 3.4.1. Inflammation related cellular events

There are certain cellular activities that are highly related to fibrosis. First and foremost is pyroptosis, a pro-inflammatory apoptosis event that lately drew attention to vascular fibrosis associated diseases (58). The creation of cell membrane holes, membrane rupture, and nuclear condensation are the primary characteristics of pyroptosis (115). These processes can be initiated by three separate signals, including inflammasome, death receptor, and mitochondrial dysfunctions (116). The molecular mechanism of pyroptosis is complicated, but the caspase family is generally considered to be involved. Caspase-1 activation and recruitment is responsive to the first signal pathway——inflammasome, which NLRP3 regulates (59). Through activation, caspase-1 cleaves pyroptosis-related molecules to their mature form, such as IL-1β, IL-18, and N-terminal fragment of gasdermin D (GSDMD) (59). Meanwhile, LPS induced caspase-4/5/11 signal as well catalyzes GSDMD (117). In vitro, those factors have been proved to be attenuated by NLRP3 inhibitor MCC950, in coronary artery inflammation model mice (59). Immunohistochemistry and western blot analysis shows that caspase-1 suppression reduces Col-1 and fibronectin expression, implying caspase-1 related pyroptosis is playing a critical role in fibrosis (58). In addition to inflammasomes, death receptor and mitochondrial dysfunctions drives caspase-3 activation, resulting in gasdermin E (GSDME) cleavage (118). The N-terminal fragment of both GSDMD and GSDME induces pore formation, which leads to pyroptosis (115). Eventually, myofibroblasts are activated by caspase-induced inflammatory cytokines release and the direct production of pyroptosis (119). This is how pyroptosis helps the progression of fibrosis. It is worth noting that some research claims SARS-CoV-2 infection also involves caspase-1-induced excessive inflammation (60). However, another research demonstrates that SARS-COV-2 has an inhibitory effect in pyroptosis (120). This inconsistency suggests pyroptosis in COVID-19 are not legible enough to be concluded unilaterally.

Furthermore, autophagy has been linked to fibrotic disorder by its regulatory influence on macrophage polarization and EndMT (61, 121). Autophagy is an essential self-degrading system in normal eukaryotic cells, for it maintains cell homeostasis by converting cellular wastes into reusable matters (62, 122). Studies have shown the inhibition of macrophage autophagy promotes M1 polarization (61, 62), which leads to the secretion of pro-inflammatory cytokines that starts ECM remodeling (63). Intriguingly, *in vitro* data shows that endothelial-specific ATG5 (autophagy-related protein 5) knockout mice exhibited EndMT-related organ fibrosis compared to littermate controls, which can be alleviated by IL-6 specific antibody neutralization (61). Although these findings were not targeted on vascular, there is no doubt that they indicated the importance of autophagy in macrophage polarization related fibrosis and in IL-6-driven EndMT. They could be the potential breach of vascular fibrosis.

#### 3.4.2. Imbalanced macrophage polarization cause chronic inflammation in vascular fibrosis

Inflammation is accompanied by both destroying and mending, and maintaining their balance is critical (62). Excessive inflammatory damage or uncontrolled tissue regeneration can both be detrimental to the healing process, while also promoting a pathological fibrotic process. By polarization and activation, macrophage is particularly connected with the above-mentioned equilibrium as well as fibrosis among all inflammation-associated cells (62). Polarization is the process by which macrophages phenotypically differentiate into two major subtypes (M1 and M2) in response to environmental signals (64). It is widely accepted that M1 is pro-inflammatory while M2 is anti-inflammatory during inflammation, echoing the previous balance of destroying and mending. In order to show an intuitionistic understanding, we highlighted the difference between M1 and M2 in Table 2.

**Table 2:** Summary macrophages in different roles in formation of fibrosis.

	M1 macrophages	M2 macrophages	References
Inflammation	Pro-inflammation	Anti-inflammation	(123, 124)
Fibrosis	Initiating inflammatory environment that begins the progression of fibrotic diseases	Excessive matrix deposition and remodeling replace the functional tissue into nonfunctional fibrotic tissue	(123)
Myofibroblasts	Activation and proliferation	Differentiation and Proliferation	(121, 124)
Fibrotic mediators	MMP-2, IL-6, ROS, CCL2	IL-10, TGF-β, Ang II, Wnt, CTGF, PDGF, galectin-3, MMP-9	(121, 125)
Fibrocyte	Recruitment of fibrocyte	Drive EndMT and FMT	(126, 127)

In early stage of inflammation, classically activated M1 phenotype initiates the tissue inflammation, and manage the removal of pathogens. To do so, stimuli such as TNF-α, IFN-γ, and LPS orient the polarization of M0 to M1, and the later further secretes pro-inflammatory molecules including IL-1β, IL-6, TNF-α, ROS, iNOS (63). These molecules can directly trigger myofibroblasts activation, meanwhile, M1 also produces chemokines (CCL2, CCL3, CXCL1, etc.) that assist fibrocytes recruitment (123, 126). In response to M1 signal, fibrocytes are recruited and migrates by the help of several chemokine receptors (CCR2, CCR3, CXCR2, etc.) (121, 123). Myofibroblasts are as well indirectly activated and proliferates (124). Furthermore, M1 produces an amount of MMP-2, which exacerbates fibrosis process through EndMT and direct degradation of ECM proteins (125).

Conversely, M2 is alternatively polarized to inhibit persistent inflammatory response (65). M2 polarization is governed by Th2 cytokines (IL-4, IL-10, IL-13, etc.) and results in fibrotic responses such as tissue remodeling (M2a), Th2 activation (M2b), and angiogenesis (M2d) (63). In order to create an environment for healing and regenerating, anti-inflammatory molecules are produced (TGF-β, IL-10, arginase, etc.) (128). However, if initial lesion prolonged, M2 exhibits a pro-fibrotic feature. As a result, multiple fibrotic molecules are produced by M2, including TGF-β, galectin-3, Ang II, Wnt, CTGF, and PDGF (63, 121, 125). The pro-fibrotic mechanisms of M2 signal involving EndMT, fibroblast-myofibroblast (FMT), cellular differentiation towards myofibroblasts, and fibrocytes proliferation (127). In addition, MMP expression was found diversely featured in M2, in comparison with M1. Scholars found that MMP-9 is overexpressed in M2, while M2 polarization results in MMP-12 elevation (129). Meanwhile, TIMP secretion accompanies. The above molecules and reactions support the fact that M2 contribute greatly to fibrosis. Nevertheless, blindly suppressing M2 polarization does not fully stand for alleviating fibrosis. Instead, persistent inflammation is why tissue repair being misled into fibrosis, therefore M1 and M2 are both heavily implicated in this process.

#### 3.4.3. Potential molecules may mediate fibrosis as inflammatory regulator

While acute inflammation facilitates healing, chronic inflammation typically implies a failure to resolve itself. While inflammation prolonged, several molecules displayed their fibrotic associated roles. Aldo, for example, can activate Th17 cells via MRs, increasing IL-17A release in adventitia (66). As a result, transcription of neutrophil gelatinase-associated lipocalin (NGAL) in fibroblasts is promoted, facilitating the elevation of gal-3, triggering collagen synthesizing and depositing (66). To further assess the fibrotic process of IL-17A, researchers conducted a study on CD4-IL-17A^ind/+^ mice, which reflect the chronic overexpression of IL-17A *in vivo*. The results are explicit, since CD4-IL-17A^ind/+^ mice exhibit an increase of serum ROS, endothelial dysfunction, and aggravated fibroblasts proliferation (67). Additionally, in CD4-IL-17A^ind/+^ mice, NO/cGMP downregulation and T-cell associated protein tyrosine kinase 2 (PYK2) upregulation were observed in contrast to control mice (67). In general, IL-17A as a critical molecule in initiating myeloid cells infiltration into the arterial wall, suppressing which has considerable potential in avoiding vascular fibrosis.

An increase in PTEN (phosphatase and tensin homolog) has been shown to be protective against Ang II-induced immune cell infiltration and vascular fibrosis. A study on sPTEN mice (transgenic mice carrying additional Pten gene) discovered its beneficial impact in reducing pro-fibrotic genes expression (Tgfb1, Col1a1, Fn1, Mmp2, etc.) triggered by Ang II (68). Evidence suggests that PTEN negatively modulates PI3K/AKT, establishing a PTEN/PI3K/AKT axis to prevent pyroptosis and apoptosis (69, 70).

Gal-3, as mentioned before, exhibit additional effect in immunoregulation (130). Although we have described its more direct influence on fibrosis, there are other mechanisms by which gal-3 affects inflammation, such as inflammatory cell extravasation regulation via binding on CD11b (Macrophages) and CD66 (Neutrophils) (71, 72). Furthermore, gal-3 enhances IL-1 and IL-18 expression by serving as a PRR and DAMP, thereby boosting the NF-κB pathway and pyroptosis (73). Nonetheless, there is currently limited evidence on gal-3 indirectly promoting fibrosis through inflammation.

#### 3.4.4. Extracellular vesicles and non-coding RNAs

Accumulating evidence suggesting non-coding RNA (ncRNA), including microRNA (miR) and lncRNA, have essential role in inflammatory and fibrosis related diseases. They can function as decoys, guides, or scaffolds in endothelial cells, contributing to the development and progression of vascular disorders (131). Meanwhile, extracellular vesicles (EVs) have been studied broadly in cardiovascular diseases, especially for their ncRNA-shipping-role in inflammatory induced fibrosis. EVs can be found in nearly every type of cell, and are composed of a lipid bilayer enclosing cargo of bioactive molecules which has significant meaning to intercellular communication (132). During atherogenesis, atherosclerosis plaque release EVs that cargos miR-23a-3p into serum, which later captured by ECs and accelerates endothelial inflammation via enhanced expression of ICAM-1, VCAM-1, E-selectin (74). Beyond that, EVs from CD4^+^ T cell and macrophage also facilitate a major part in pro-fibrosis. For example, macrophage derived EVs carrying with lncRNA GAS5 can lead to EC apoptosis, in response to excessive lipoproteins accumulation (75). Through targeting miR-138-5p/RhoC axis, EVs from macrophages show pro-fibrotic effect by transmitting circUbe3 (76). Furthermore, activated CD4^+^ T cells secretes EVs loading miR-142-3p, to contribute to fibrotic remodeling after myocardial infraction, via activation of myofibroblasts (77).

However, the significance of ncRNA goes beyond. miR-21 is abundantly expressed in mammalian cells, and is known for their anti-inflammatory effect. Novel findings show that miR-21 have essential role in regulating inflammation in ECs and VSMCs, targeting PPAR-α (78). miR-21 is also involved in cardiac fibrosis formation via TGFβ1/Smad3 pathway (79). Overexpressed miR-122 are found suppressive to PTEN, leaving an indication that miR-122 participates in pyroptosis. Similarly, studies found miR-122 knockdown alleviates apoptosis, inflammation, and OS (80), which suggests a viable target against fibrosis. miR-126 has special role in angiogenesis via VEGF signal activation. In addition, miR-126 displays certain anti-inflammatory feature, since its deficiency in EC results in TNF-α-induced VCAM1 expression, which mediates leukocyte adherence to EC (133). lincRNA-p21 is downregulated in atherosclerosis mice and patients with coronary artery disease, silencing which leads to VSMC and macrophage proliferation, yet apoptosis repression (78). LEENE represents for lncRNA that enhances endothelial nitric oxide synthase (eNOS) expression. LEENE works as an enhancer to mRNA promoter, which facilitates EC inflammation suppression (134). The lncRNA myocardial infarction associated transcript (MIAT) is upregulated in diabetic patient monocytes, and decreased in senescent fibroblasts. MIAT overexpression has shown relevance in high-glucose induced apoptosis, and diabetic pathological angiogenesis, reversing inhibition to VEGF by decoying miR-150 and miR-200a (131).

In summary, inflammation, in its diverse aspects, has enormous influence and closely related to vascular fibrosis. Deepening its understanding would be a major breakthrough in related pathological conditions.

### 3.5. Other mediators in vascular fibrosis

We have so far elaborated the molecular mechanisms of vascular fibrosis from several different aspects including RAAS and TGF-β, mitochondria, EndMT, as well as inflammation. However, there are several more factors closely related to vascular fibrosis that cannot be overlooked.

Marinobufagenin (MBG), an endogenous cardiotonic steroid being identified in mammalian extracellular fluids, promotes vascular fibrosis by inhibiting friend leukemia virus integration 1 (Fli-1) along with activating TGF-β1/Smad pathway (81). Since Fli-1 function as a nuclear inhibitory transcription factor to Col-1 gene, MBG elevation results in Col-1 overexpression (82). Interestingly, spironolactone has shown alleviation to the profibrotic effect of MBG (83). ROCK (Rho-associated coiled-coil forming protein kinases), which expresses in most vascular cells (ECs, VSMCs, fibroblasts, macrophages, and lymphocytes), has been shown to mediate cellular migration, adhesion, polarity, and cytokinesis via phosphorylating downstream cytoskeleton mediator proteins (84). ROCK suppresses eNOS (NO generation), promotes EndMT, switches VSMCs to a migratory, proliferative phenotype, and regulates M1/2 alternation in macrophages (84). Moreover, ROCK promotes inflammation and OS, since the upregulation of IL-6, TNF-α, IFN-γ, and SOD is accompanied with ROCK overexpression in sepsis models (85). Nonetheless, several drugs have been reported to be ROCK-resistant. Statins, for instance, can suppress RhoA/ROCK via competing TGF-β1/Smad activation, as well as p38-MAPK (86). A study has shown that PAI-1 (plasminogen activator inhibitor-1) mutant mice have higher levels of Col-1 expression and coronary perivascular fibrosis (87). Intriguingly, apelin, an endogenous peptide expressed by ECs, has the capacity to block Ang II induced collagen and MMP-2 expression as well as PAI-1-induced Rho kinase activation, indicating a role in anti-fibrosis (88).

## 4. Age and sex brought heterogeneousness to diverse fibrotic responses

Before putting clinical disorders into discussion, it is important to know that age and sex play a vital role in individual responses. When it comes to vascular fibrosis, age and sex exhibits unique characteristics in immune system and inflammation, collagen deposition, and eventually, fibrosis development (135, 136). The concept of those certain aspects being influenced by aging and sex will be generally explored in this section.

### 4.1. Age and sex in immune system and inflammation

Gender and age perform substantially in the immune response, influencing susceptibility to infections, vaccine response, and function in immune system (137). Aging is linked to the occurrence of long-lasting inflammation as well as a general decrease in immunological function (138).

First of all, there are certain variance in immune cells: NK cells are observed to be higher in male, while plasma cell levels are higher in female (139). The mechanisms within could be related to the prohibit effect of estrogen and progesterone on NK cells (140). Meanwhile, female exerts more activated T cells and B cells than male (141). These differences are claimed to be more significantly impacted through aging. Meanwhile, some research suggests aging to be linked with the increase in monocytes, decrease in T cells, and expression of PBMCs inflammatory markers (142).

Secondly, gene and molecular expression impacts the bias of gender and age difference in immune system. Aging influences pro-inflammatory genes expression, which is severer in male (138). According to recent studies, male experience more IFN-γ receptor activation and MAPK signal induced inflammations (139). They also discovered ZFP36 and DUSP2 are two genes that have the highest level in elder male, which represents weaker adaptive immunity, due to T cell inhibition (139). In addition, we have mentioned a more active T and B cell in female, which could be related to upregulated BAFF/APRIL system in women. While BAFF manages the viability of B cells, APRIL controls cellular proliferation which are essential in humoral immunity (139). Unfortunately, BAFF and APRIL levels lead to their increased risk in autoimmune diseases (143).

Lastly, male and female, young and elder, have distinct sensitivity in inflammation or infection. Aging is linked to the development of chronic inflammation as well as a general decrease in immunological function. Some research suggests that the innate immune system of elderly females is more prone to inflammation than that of elderly males (137). However, women have an enhanced immune response to viral infections than men, resulting in quicker pathogen clearance and increased vaccination effectiveness in females (144). For instance, men have a higher susceptibility to several infectious illnesses, as well as a higher fatality rate (139). After the age of 65, men and women's immune system cells differed the most (141). Older female functions slower in acute reaction or sickness yet shows resistance to certain long-term infections (141). Older men, on the other hand, exhibited higher levels of activity in the innate immune system, the body's generic but faster-reacting defense force (145).

### 4.2. Age and sex in collagen deposition

Age as well as gender may additionally impact collagen deposition in different tissues. Increased collagen and fibrin deposition occurred in elderly normal controls when compared to non-elderly controls, and this was even more pronounced in elderly patients with nasal polyps (146), which was further confirmed by studying model organisms such as mice and C. elegans (147). The deposition of collagen fibers after surgery is significantly reduced in older men but not in women (148). These results imply that age and sex can have a major effect on collagen deposition throughout different tissues and that analyzing these differences is vital for developing potent therapies for age-related diseases.

### 4.3. Age and sex in fibrosis development

It is eventually our consideration of fibrotic diseases for any heterogeneousness that gender and age may cause. Age-related alterations to the cardiac ECM differ by gender, with elderly male hearts being more fibrotic than female hearts (149). Gender-dependent cardiac fibrosis occurs with age in rats, but the underlying processes remain unknown (150). In those with atrial fibrillation, increasing age and female sex are correlated with a higher incidence of atrial fibrosis (151). We should additionally pay close attention to the fact that the occurrence of greater fibrosis in adult skeletal muscle cells increases dramatically with age, which is associated with TGF-β signaling (152). Similarly, it has been presented that female hormones can ameliorate the advancement of cystic fibrosis in the lung, and there is substantial evidence that estrogen plays an essential role in the development of cystic fibrosis (153). One issue that cannot be overlooked is that aging and diabetes are substantial risk factors for the development of penile fibrosis (154). The above findings emphasize the intricate relationships between age, gender, and the genesis of fibrosis in diverse tissues and disorders. These distinctions can aid the development of tailored and effective fibrotic treatments.

## 5. Vascular fibrosis correlated diseases and possible interventions: bench to bed

**Figure 3:**
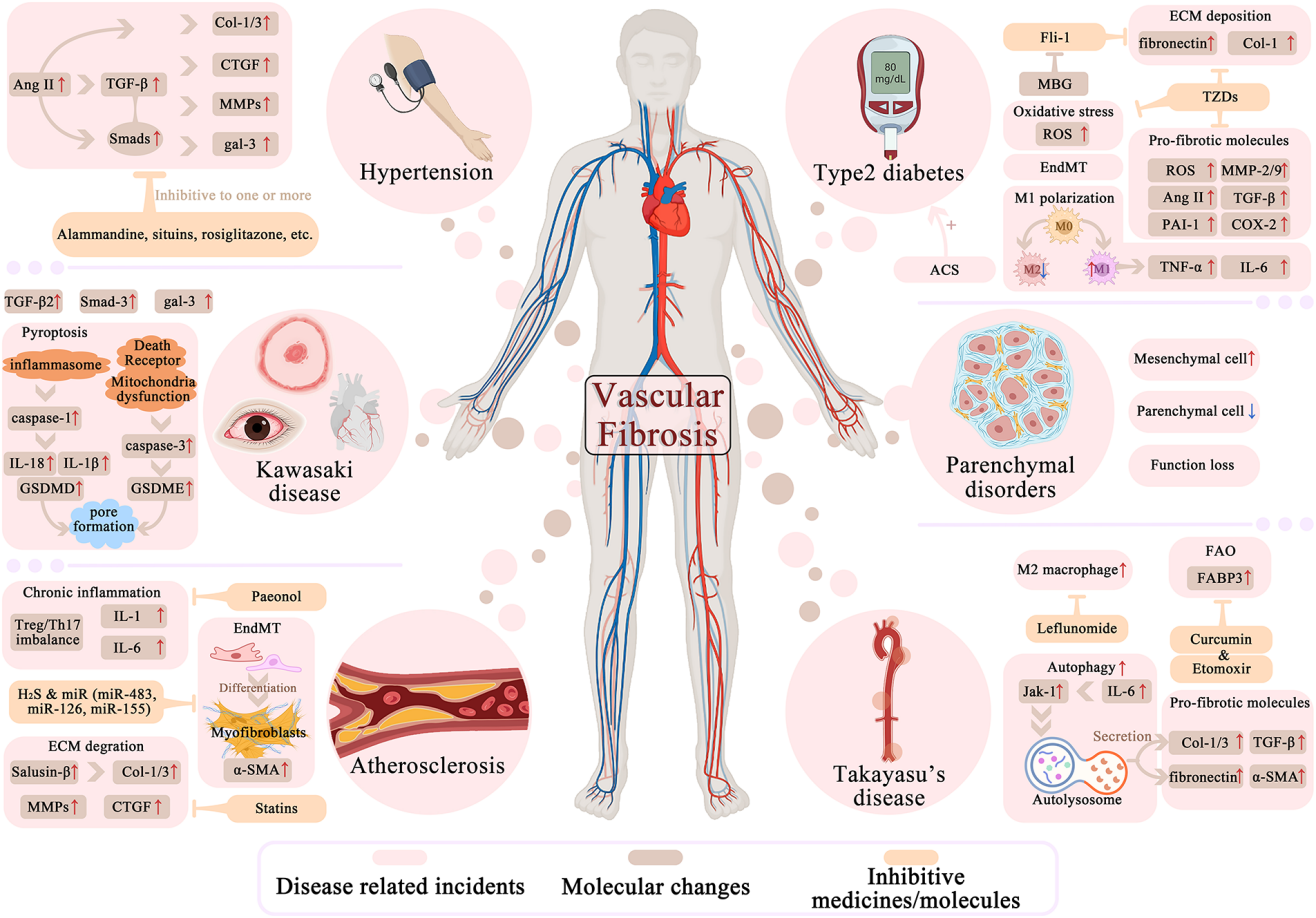
Molecular and cellular factors in vascular fibrosis related diseases and potential interventions. Hypertension, atherosclerosis, Kawasaki disease, Takayasu's diseases, diabetes and parenchymal disorders are all linked to vascular fibrosis in certain degree and are generally elaborated in this part. In different color mark different aspects of disease related fibrosis process: For columns in light red list the fibrotic-related major cellular or biologic incidents that undergoes in pathogenesis. Brown is for the diverse molecules with arrows indicating their up/down-regulations during diseases. Yellow is for several inhibitive medicines or molecules that can reverse the fibrotic molecules or events. The process of pyroptosis, autophagy, EndMT are also shown concisely.

Fibrosis is a widely identified vascular condition that develops progressively and is linked to aging and hypertension. Understanding the processes behind vascular fibrosis may lead to the development of new treatment strategies, as vascular involvement in fibrosis pathophysiology is an area of ongoing research. However, there is currently no cure for vascular fibrosis, except there are some interventions that can manage the condition. In this section, we just highlight the associated disorders as well as provide our evaluation in order to establish a novel therapeutic approach for future clinical practice (Figure 3 for details).

### 5.1. Hypertension

Hypertension is the leading cause of morbidity and mortality worldwide, and is associated with an increased risk of cardiovascular disease. Normally, the heart ejects blood, and the arcus aortae dilate and buffer the systolic and diastolic pressures as they pass to provide appropriate tissue perfusion (155). This mechanism is commonly accepted to be strongly dependent on artery elasticity and compliance, whereas vascular fibrosis-induced arterial stiffness lacks this feature (1). It is especially unsettling since hypertension being a direct outcome of arterial remodeling and fibrosing, may be easily obtained through collagen deposition (36). The expression and proliferation of VSMCs can also lead to the thickening of the vessel wall, which enhances transverse aortic contraction and blood pressure (48).

There are several medications that alleviate hypertension through various methods, some of which grabbed our attention due to their anti-fibrosis properties. Firstly, alamandine can reduce blood pressure by preventing Ang II-induced arterial remodeling (36). Secondly, statins, as the inhibitors of the 3-hydroxy-3-methylglutaryl coenzyme A (HMG-CoA) reductase, have been found to be pleiotropically beneficial against fibrosis and hypertension through inhibiting TGF-β/Smad (86), in addition to its well-known cholesterol-lowering activity. Their anti-fibrotic role leaves us a novel target for resistant hypertension.

Pulmonary arterial hypertension (PAH) is one particular hypertension in the lungs, defined as an ≥20 mmHg resting pulmonary arterial pressure in mean (156). Pulmonary illness and dyspnea are inseparable from PAH, partly due to pulmonary artery smooth muscle cell (PASMC) proliferation, inflammation, and severe fibrosis (73). Scholars show special interest in their latest discovery in a cluster of PAH-related mediators, including gal-3, StAR, caspase-1, and programmed death ligand-1 (PDL-1). PDL-1, along with caspase-1, is implicated in pyroptosis, which is associated with the production of fibrosis and PAH (58). Furthermore, the research found that NEDD9, a TGF-β independent mediator, can trigger fibrosis in PAH via oxidative modification at Cys 18 (157).

### 5.2. Vascular disorders

Atherosclerosis, one of the leading causes of CVD, is characterized by impaired degradation of ECM (collagen and fibronectin), VSMCs proliferation, as well as calcification (49), therefore lies connection with fibrosis. EndMT has been widely discovered in atherosclerosis due to its promotive effect in plaque formation. EndMT generates mesenchymal cells that produce pro-inflammatory cytokines, ECM proteins, and MMPs (158). As research deepens, certain chemicals strongly linked to atherosclerosis have been discovered. For example, gal-3 overexpresses in plaque, salusin-β TGF-β-dependently promotes Col-1/3 expression, and ncRNAs (miR-125b, miR-21, Let-7c, Let-7g) generally expresses in the thickened vascular wall (35, 49, 103). Intriguingly, a study on a natural substance paeonol revealed that it has a role in atherosclerosis suppression by enhancing gut microorganisms' short-chain fatty acids (SCFAs) synthesis to modulate Treg/Th17 balance in the spleen, hence preventing vascular fibrosis (159). Statins also alleviates atherosclerosis by down-regulating the expression of Ang II-induced profibrotic factors and ECM proteins (86). Furthermore, H_2_S and specific microRNAs (miR-483, miR-126, miR-155) have a certain role in alleviating vascular fibrosis and atherosclerosis. The experimental evidence shown above illustrates that the occurrence, progression, and therapy of atherosclerosis is a tremendously complicated process involving different routes and cytokines or active substances.

As mentioned above, vascular inflammation is highly associated with fibrosis. Takayasu's arteritis (TAK) is a chronic inflammatory disease characterized by granulomatous vasculitis and fibrosis in the aorta and its branches (47). Perfusion is hampered by persistent inflammation and fibrosis, which leads to organ failure and death (160). Multiple fibrotic biomarkers, including TGF-β, Col-1/3, fibronectin, and α-SMA, have been found elevated in TAK arteries than in normal arteries (161). Unfortunately, conventional therapies with glucocorticoids and immunosuppressants can only stabilize inflammation, but behaved badly against existing fibrosis-induced structural damage. Research has revealed that vascular fibrosis in TAK is promoted by IL-6 related autophagy via JAK1/STAT3 pathway, implying impeding autophagy, as well as IL-6 inhibitor, may be beneficial in the treatment of TAK (161, 162). Leflunomide (LEF), an anti-inflammatory medication, has been demonstrated to diminish TAK by blocking and lowering macrophage subtype M2 rather than M1 (160). Another study found that Curcumin and Etomoxir exhibit properties of anti-fibrosing, targeting FABP3 to modulate FAO in AAFs as a possible treatment for TAK and other vascular fibrosing conditions.

Furthermore, vascular fibrosis is found in Kawasaki disease (KD), a well-documented but poorly understood systemic pediatric vasculitis characterized by multisystem inflammatory syndrome (MIS), severe toxic shock syndrome, and coronary artery aneurysm (163, 164). In KD model mice, coronary artery inflammation pyroptosis-related factors (NLRP3, caspase-1, IL-1β, IL-18) exhibited a significant upregulation in ECs (59), showing EC pyroptosis participates in KD. Intriguingly, SARS-CoV-2 was identified to coincide with KD in MIS-C degree (163, 165), hence understanding the mechanisms of SARS-CoV-2 may be the potential key to unmasking KD. In addition, studies have demonstrated that gal-3 and TGF-β/Smad levels are much higher in KD patients, making them appealing therapeutic targets for KD and fibrotic dysfunction (166, 167).

### 5.3. Diabetes and endocrine disorders

Evidence has confirmed that there is a linkage between vascular fibrosis and endocrine disturbance. Diabetes, particularly type 2 diabetic mellitus (DM2), that shares consistency with vascular fibrosis according to multiple studies. On one hand, fibrosis directs DM: Experiencing ECM deposition, cytoarchitectural abnormalities, microvascular dysfunction, and impaired endocrine cell-blood exchanges in pancreatic islets lead to aberrant insulin secretion (168). Cell type modifications, such as the conversion of pericytes to fibroblasts and EndMT, are critical in triggering DM2 vascular fibrosis (49, 168). DM is also linked to autonomous cortisol secretion (ACS) in individuals with primary aldosteronism (PA) (169). PA patients with ACS had a considerably greater diabetes incidence and a larger fibrosis area in blood vessels, indicating that an ACS show-up implies poor vascular remodeling (169). On the other hand, DM2 lures fibrosis in aortic or other cardiovascular tissue: developing DM boosts myofibroblasts activation, inhibits macrophage M2 polarization, induces a profibrotic phenotype in immune cells, increases ROS, and initiates EndMT (81, 170, 171). Interestingly, various fibrotic-associated molecular alterations were observed in salt-loaded DM2 mice, including increased TGF-β, Col-1, and fibronectin, as well as decreased Fli-1, which could be reversed by MBG (81, 83). In salt-loaded DM2 patients, experiencing a Na^+^–K^+^–ATPase malfunction might be the cause of vascular injury and remodeling. MBG, as its inhibitor, contributes to this pathological condition by suppressing it, therefore benefiting DM2 (81). In addition, rosiglitazone as a classic drug on insulin resistance DM2 patient, have attached repressive impact on Ang II-induced AT-1R, IL-6, and TNF-α elevation (172), as well as VSMCs and myofibroblasts proliferation (173, 174). These changes vastly benefit vascular fibrosis. Another study indicates rosiglitazone, as a PPAR-γ activator, benefits in DM2 by lowering CTGF expression and ECM deposition in Ang II-induced fibrosis (175). However, along with other thiazolidinediones (TZDs), rosiglitazone may be associated with severe side effects including heart failure and pulmonary edema (176), therefore its clinical application is restricted and must be paid particular attention. Nevertheless, many research suggest rosiglitazone attenuative to serum level of fibronectin, TGF-β, PAI-1, and inflammatory marker (MMP-2, MMP-9, COX-2) in DM2 patients (177, 178). Based on the evidence presented above, we may deduce that there is a pertinent association between diabetes mellitus and vascular fibrosis, and that this link impacts the prognosis and progression of the disease, while also providing a novel target for vascular fibrosis treatment.

### 5.4. Parenchymal organ lesions

Since myocardial fibrosis is frequently coupled with vascular fibrosis, both molecular mechanisms and pathological processes are intertwined. Chronic hypertension and arteriosclerosis increase cardiac afterload, leading to myocardial hypertrophy, coronary heart disease, heart failure, and other complications. In the aging kidney, T and B cells, together with resident fibroblasts, form tertiary lymphoid tissues that cause uncontrollable inflammation and delay tissue repair (179). Furthermore, severe fibrosis of small blood vessels can cause parenchymal tissues and cells to grow, leading to fatal diseases including liver cirrhosis and brain fibrosis (58). However, fibrosis is not a one-way road. To slow the process of organ fibrosis, ECM protein degradation, chronic inflammation regression, tissue injury resolution, and myofibroblasts deactivation are all required (180). Currently, a number of anti-fibrosis medications are being tested in clinical trials. Nintedanib is an antifibrotic drug licensed for patients with idiopathic pulmonary fibrosis (IPF) by blocking several tyrosine kinases implicated in fibrosis development (181). Another anti-fibrotic medicine, Pirfenidone, has been licensed for use in individuals with IPF to decrease disease development by reducing the production of pro-fibrotic cytokines and growth factors (182). It should be emphasized that lowering oxidative stress can also help slow or stop fibrosis progression (183). As previously stated, some immune modulation is involved in the genesis of fibrosis (184).

## 6. Limitation and challenge

Despite numerous experimental research on vascular fibrosis, it remains a limitation when it comes to clinical trials and treatments (185). To begin with, although several medications have been explored for fibrosis therapy, there are presently no viable therapeutics for vascular fibrosis. This emphasizes the importance of additional research to uncover novel treatment targets (186). The second and most essential reason is that the processes of vascular sclerosis are still poorly understood. Although the processes of vascular fibrosis have been established, the fundamental mechanisms are still poorly understood (186). The third issue that can't be overlooked is the lack of a reliable biomarker for vascular fibrosis diagnosis and monitoring (187). Finally, despite several clinical cohort studies that have been conducted, there is currently a paucity of viable treatments for vascular fibrosis (188). Many anti-fibrosis medicines have been tested exclusively on animals and have shown promising results, but the transition from bench to bed remains a lengthy time. Based on the facts stated above, we have reason to believe that there are still particular challenges that will necessitate collaboration between researchers, doctors, and clinical trials in the treatment of vascular fibrosis.

## 7. Conclusion

Fibrosis affects almost all tissues in the body. Although fibrosis is frequently associated with strong inflammatory response, growing studies indicate that there are particular mediators and pathways contributing to the pathophysiology of fibrosis that are differ from the inflammatory processes. We must first recognize this distinction before developing effective therapies for fibrotic illness. In this review, we emphasized the underlying changes in structure and function in vascular fibrosis, concluded some recently discovered or solid mediators, and illustrated a few related ailments with prospective interventions. Vascular fibrosis, characterized by ECM deposition, VSMC proliferation, hindered matrix breakdown, and EC dysfunction, causes numerous dysfunctions in cardiovascular and other systems via RAAS, TGF-β, MMPs, and other linked mediators. Targeting on these mediators may be of valuable means in discovering related treatments. Despite decades of research in vascular fibrosis, therapeutic drugs have always been few or ineffective, especially to pre-existing fibrosis. Due to the complexity and diversity of the vascular system, it is difficult for a single element to be successful and target-specific in the overall rehabilitation of vascular fibrosis. This is why we believe the understanding presented in this review would be especially beneficial to researchers in developing practical and therapeutic medicines or methods for vascular fibrosis-related disorders.
